# Assessing Rodent-Induced Ecological Disturbance in Natural Grasslands Using Multi-Source Spatial Data

**DOI:** 10.3390/ani16142260

**Published:** 2026-07-21

**Authors:** Miaomiao Huang, Qiqige Wulan, Ting Wang, Liqing Wang, Yuchuang Hui, Rui Hua, Limin Hua

**Affiliations:** 1 Institute of Grassland Research, Chinese Academy of Agricultural Sciences, Hohhot 010010, China; huangmm0306@163.com (M.H.); wangliqing0471@163.com (L.W.); huiyuchuang6118@163.com (Y.H.); 2 Key Laboratory of Biohazard Monitoring, Green Prevention and Control for Artificial Grassland, Ministry of Agriculture and Rural Affairs, Inner Mongolia Key Laboratory of Grassland Conservation Ecology, Hohhot 010010, China; 3 Engineering and Technology Research Center for Alpine Rodent Pest Control of National Forestry and Grassland Administration, Key Laboratory of Grassland Ecosystem of the Ministry of Education, College of Grassland Science, Gansu Agricultural University, Lanzhou 730070, China; wangting921221@163.com; 4 Biosafety Protection Center, Forestry and Grassland Bureau of Dameng Banner, Baotou 014000, China; dmqsfz@163.com

**Keywords:** plateau pika, Brandt’s vole, multi-source spatial data, rodent damage

## Abstract

High-density rodent populations cause severe habitat degradation and ecological imbalance in natural grasslands through intense foraging and burrowing activities. However, dynamically monitoring these small mammals and assessing their large-scale damage using traditional ground surveys alone is challenging. In this study, we evaluated rodent damage severity in alpine meadows and typical steppe by proposing an integrated framework that combines ground, unmanned aerial vehicle (UAV), and satellite data. Using data from 36 plots per grassland type, we extracted a suite of ecological parameters, including aboveground biomass, vegetation cover, community height, rodent burrow density, and plant diversity metrics, to construct a plot-scale Rodent Damage Index (RDI). This RDI was then linked with a satellite-derived Remote Sensing Ecological Index (RSEI) to model and map damage severity at the regional scale. Separate linear regression models were developed for the two grassland types. The alpine meadow model exhibited better model fit and predictive performance (fitting $R^{2}$ = 0.762, RMSE = 0.136; LOOCV $R^{2}$ = 0.729, RMSE = 0.145, 95% CI: 0.580–0.886) than the typical steppe model (fitting $R^{2}$ = 0.574, RMSE = 0.198; LOOCV $R^{2}$ = 0.478, RMSE = 0.214, 95% CI: 0.279–0.787), highlighting that the predictive relationship model performance differs significantly between grassland types. Our findings demonstrate that integrating multi-source, cross-scale spatial data is an effective approach for assessing rodent damage. Furthermore, these results indicate that rodent damage assessment should be grassland-type-specific to ensure accuracy and support targeted rodent damage management planning.

## Simple Summary

Rodent damage is one of the key biotic factors driving grassland degradation, affecting vegetation growth and ecosystem stability. This study combined ground surveys, unmanned aerial vehicle (UAV) imagery, and satellite remote sensing to assess rodent damage severity in alpine meadows and typical steppe. By integrating ecological indicators across multiple spatial scales, we produced regional maps of rodent damage severity. The results showed that grassland type strongly influenced assessment accuracy, with better performance in alpine meadows than in typical steppe. These findings provide a practical approach for large-scale rodent damage monitoring and can support more effective rodent management and grassland restoration.

## 1. Introduction

Natural grasslands are a vital component of terrestrial ecosystems and play a critical role in maintaining ecological security, ensuring sustainable livestock production, and protecting biodiversity. They also provide important habitats for numerous wildlife species, including herbivores, birds, insects, and small mammals, many of which are closely associated with grassland ecosystem structure and function [1]. Alpine meadows on the Qinghai–Tibet Plateau and typical steppe in Inner Mongolia are two representative natural grassland types with important ecological and productive value. In terms of productive value, grasslands provide natural forage resources for grazing livestock and support the production of meat, milk, wool, and other livestock products, thereby sustaining pastoral livelihoods and regional animal husbandry economies [2,3]. Alpine meadows are a key part of the ecological security barrier on the Qinghai–Tibet Plateau and play an important role in water conservation, climate regulation, and biodiversity protection [4,5]; typical steppe is a major grassland type in northern China and supports livestock production and regional ecological balance [6]. In recent years, rodent damage has become increasingly severe in both grassland types due to climate change, grazing pressure, and grassland degradation, making it a major concern for grassland conservation and management [7,8].

Rodent species and their associated damage characteristics differ among grassland types [9]. In alpine meadows, the plateau pika (*Ochotona curzoniae*) acts as a dominant disturbance agent. High pika densities can reduce vegetation productivity, alter surface conditions, and increase the risk of grassland degradation [10]. Its damage is mainly associated with burrowing, soil mounds, bare-patch expansion, and sward fragmentation [11]. However, although the plateau pika is also considered a keystone species in alpine ecosystems, with potential roles in food web maintenance, nutrient cycling, and habitat heterogeneity, its ecological effects are complex and context-dependent [12]. In a typical steppe, Brandt’s vole (*Lasiopodomys brandtii*) is a dominant rodent species associated with grassland disturbance. Population outbreaks can substantially reduce vegetation cover, community height, and forage availability through burrowing and foraging activities, thereby weakening grassland productivity and livestock carrying capacity in northern grasslands [13]. Compared with plateau pika disturbance, Brandt’s vole disturbance is more strongly influenced by precipitation variation, food availability, and outbreak dynamics (rapid and often synchronous increases in rodent population density across affected areas) and is mainly expressed through plant consumption, forage loss, and changes in plant community structure [14,15]. Overall, rodent damage in alpine meadows is characterized mainly by surface disturbance, whereas damage in typical steppe is more strongly related to vegetation consumption [16,17]. These differences suggest that rodent damage is shaped by both species identity and grassland type. Therefore, a single general model is unlikely to capture the variation in damage characteristics, and species- and grassland-type-specific monitoring models are needed.

The management of grassland rodent damage should follow a prevention-oriented approach and adopt targeted measures based on damage severity. The prerequisite for achieving this goal is the establishment of an efficient, accurate, and dynamic monitoring system. For a long time, the assessment of rodent damage severity in grasslands has relied mainly on conventional ground surveys [18]. Although ground surveys provide reliable field observations, they are time-consuming, labor-intensive, and limited in spatial coverage, making them unsuitable for rapid and large-scale monitoring of natural grasslands [19]. In many areas, limited monitoring resources and a lack of trained personnel further reduce the efficiency of traditional survey methods [20]. Remote sensing offers a practical solution to these limitations. Ground surveys can provide accurate sample data for model calibration, while UAV imagery can capture fine-scale damage features such as burrow density and small vegetation patches [21]. Satellite remote sensing, with its broad coverage and repeated observations, is well-suited for regional monitoring of grassland condition and the spatial distribution of rodent damage [22]. Although remote sensing has increasingly been used in grassland degradation and rodent damage studies, important gaps remain in the cross-scale assessment of rodent disturbance. Existing UAV-based studies have mainly emphasized the detection of fine-scale surface features, such as burrow entrances, bare soil patches, and vegetation cover, whereas satellite-based studies have generally focused on broader patterns of vegetation degradation or ecological condition [23,24]. However, these two scales are often treated separately, and the link between plot-scale ecological damage and regional-scale remote sensing indicators has not been fully established. Moreover, rodent disturbance mechanisms differ between alpine meadows and typical steppe: plateau pika disturbance is mainly expressed through burrowing and surface fragmentation, whereas Brandt’s vole disturbance is more closely associated with vegetation consumption and forage loss [13,25]. As a result, a single generalized remote sensing model may not adequately capture rodent damage severity across different grassland types. Addressing this gap requires an integrated framework that combines ground observations, UAV-derived disturbance indicators, and satellite-based ecological indices. Together, these data sources provide a basis for developing a multi-source monitoring framework for rodent damage assessment.

To address these research gaps and overcome the spatial limitations of conventional field surveys, we developed an integrated framework combining ground observations, UAV imagery, and satellite remote sensing. The framework was applied to alpine meadows and typical steppe, two representative natural grassland types characterized by distinct dominant rodent species and disturbance patterns. RSEI was selected instead of a single vegetation index, such as NDVI, because rodent disturbance can simultaneously affect multiple components of surface ecological conditions. Rodent activity may reduce vegetation greenness and cover, increase bare soil exposure, alter surface moisture, and modify land surface temperature [26,27]. Given the limited number of paired plot-scale RDI and satellite-derived RSEI samples and the need for an interpretable model for regional mapping, linear regression was selected as a parsimonious linking approach. This approach allows the direction and strength of the RDI–RSEI relationship to be clearly quantified, while reducing the risk of overfitting that may occur when more flexible machine-learning models are applied to small datasets. We hypothesized that rodent damage would alter local surface conditions, including vegetation cover, surface temperature, and soil moisture, and that these changes could be quantified at the plot scale and extrapolated to the regional scale. Specifically, we aimed (1) to construct a plot-scale Rodent Damage Index (RDI) by combining ground surveys and UAV imagery to extract indicators such as rodent burrow density, aboveground biomass, vegetation cover, community height, and plant diversity; (2) to derive a satellite-based Remote Sensing Ecological Index (RSEI) from Landsat 8 data as a potential predictor; and (3) to establish a predictive model linking RDI and RSEI for regional mapping of rodent damage severity. This study provides a practical basis for rapid monitoring and assessment of rodent damage in fragile grassland ecosystems.

## 2. Materials and Methods

### 2.1. Study Area

**Figure 1:**
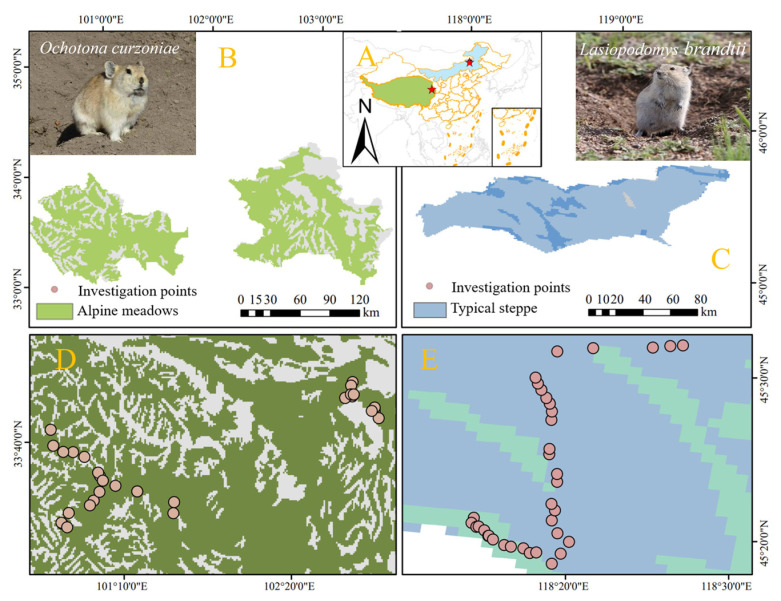
Map of the Study Area (**A**) Location of the study area in China, (**B**) Study area of alpine meadow on the Qinghai–Tibet Plateau, (**C**) Study area of typical steppe in the Inner Mongolia Autonomous Region, (**D**) Sampling sites for plateau pika (*Ochotona curzoniae*), (**E**) Sampling sites for Brandt’s vole (*Lasiopodomys brandtii*). The asterisk in (**A**) indicates the locations of the study areas shown in (**B**,**C**).

The alpine meadow study areas were located in Jiuzhi County, Qinghai Province, and Ruoergai County, Sichuan Province, on the eastern Qinghai–Tibet Plateau, whereas the typical steppe study area was located in Dongwuzhumuqin Banner, Inner Mongolia, northern China (Figure 1). The Jiuzhi study area has a typical plateau continental climate and an average elevation above 4000 m a.s.l., with elevations across the county ranging from approximately 3568 to 5369 m a.s.l. The mean annual temperature is approximately 0.1 °C, and the mean annual precipitation is about 764 mm, most of which occurs from May to September. The Ruoergai study area is located at an average elevation of approximately 3500 m a.s.l., with a mean annual temperature of 0.6–1.1 °C and annual precipitation of approximately 500–600 mm. Owing to the high elevation and low temperatures, the alpine meadow region has a short growing season that is largely restricted to the warm and wet period and is characterized by a short or absent frost-free period. The plant communities are mainly dominated by *Kobresia pygmaea*, *Elymus nutans*, *Anemone trullifolia*, *Potentilla anserina*, *Gentiana formosa,* and *Thalictrum alpinum*. Field surveys indicated that the plateau pika (*Ochotona curzoniae*) was the dominant rodent species in the alpine meadow study areas.

In contrast, the typical steppe study area in Dongwuzhumuqin Banner is characterized by a lower elevation and a temperate continental semi-arid climate, with relatively low precipitation and strong evaporation. Meteorological records indicate an elevation of approximately 838.7 m a.s.l., a mean annual temperature of about 2.48 °C, and mean annual precipitation of approximately 234.23 mm. The growing season generally extends from early May to late September, during which most vegetation growth and precipitation occur. The plant communities are mainly composed of *Stipa grandis*, *Leymus chinensis*, *Cleistogenes squarrosa*, *Allium polyrhizum*, and *Neopallasia pectinata*. Field surveys indicated that Brandt’s vole (*Lasiopodomys brandtii*) was the dominant rodent species associated with visible burrow disturbance in the typical steppe during the survey period. Species identification was based on field observations, burrow characteristics, and regional identification keys.

### 2.2. Data Acquisition and Processing

#### 2.2.1. Ground Data Acquisition and Processing

During the peak growing season, 36 plots (50 m × 50 m) were selected in each grassland type to cover a visible gradient of rodent damage severity. The apparent differences in damage severity were identified during preliminary field reconnaissance based on burrow entrance density, exposed soil patches and visible reductions in vegetation cover and community height. These visual differences were later quantified using ground-based measurements and UAV-derived indicators. Ground monitoring indicators were selected to quantify visually apparent rodent disturbance features observed during preliminary field reconnaissance and detectable from UAV imagery, including burrow entrances, exposed soil patches, vegetation fragmentation, reduced vegetation cover, lower community height, and reduced aboveground biomass. Although plateau pika and Brandt’s vole have different disturbance mechanisms, these indicators capture common and comparable vegetation and surface responses to rodent disturbance. Therefore, aboveground biomass (AGB), vegetation cover (VC), community height (CH), and rodent burrow density (RBD) were measured as ground monitoring indicators.

Vegetation cover was measured using a step-point method along a diagonal transect within each 50 m × 50 m plot. One diagonal transect was established from one corner of the plot to the opposite corner. During the survey, the observer walked along the transect at a normal pace, and the toe tip at each step was used as a point-intercept observation point. Each point was recorded as either vegetation-covered ground or bare ground. Plot-level vegetation cover was calculated as the proportion of vegetation-covered points relative to the total number of observation points. To reduce observer bias, all vegetation cover measurements were conducted by the same trained survey team using a consistent protocol [28]. Community height was measured by randomly selecting 10 dominant plant individuals within each plot and measuring their height using a steel tape; the mean value was used to represent plot-level community height [29]. Aboveground biomass was measured independently using three 0.5 m × 0.5 m subplots placed at equal intervals along the same diagonal transect. All aboveground plant material within each subplot was clipped at ground level, dried at 65 °C to constant weight, and weighed. The mean biomass of the three subplots was used as the plot-level AGB [30]. Because rodents are elusive and difficult to observe directly, burrow density was counted in each plot and used as a proxy for relative rodent abundance. Plant community diversity was included because rodent disturbance may affect not only vegetation biomass, cover, and height, but also species composition and community structure. Two complementary diversity indices were used in this study. The Shannon–Wiener diversity index ($H^{\prime }$) reflects both species richness and the relative distribution of individuals among species, and is therefore useful for characterizing overall plant community diversity. The Margalef index (D) mainly reflects species richness after accounting for the total number of individuals, and provides additional information on changes in species richness caused by rodent disturbance. Therefore, using both indices allowed the RDI to incorporate different aspects of plant community response to rodent damage. Based on the vegetation survey data, the Shannon–Wiener diversity index ($H^{\prime }$) and the Margalef index (D) were calculated for each plot using the following formulas:

(1) $$H^{\prime }=-i=-\sum_{i=1}^{S}Pi\ln Pi\;\;$$

where $H^{\prime }$ is the Shannon–Wiener diversity index; S is the total number of species; $P_{i}$ is the relative abundance of species i

(2) $$Pi=\frac{Ni}{N}$$

where Ni is the number of individuals of the i-th species; N is the total number of individuals across all species

(3) $$D=\frac{S-1}{\ln N}$$

where D is the Margalef diversity index; S is the number of species; N is the total number of individuals.

#### 2.2.2. UAV Image Acquisition and Processing

UAV surveys were conducted simultaneously with ground investigations in each plot. Imagery was acquired using a DJI Mavic 2 Pro UAV (SZ DJI Technology Co., Ltd., Shenzhen, China) equipped with a Hasselblad L1D-20c RGB camera. Flights were conducted under clear and windless conditions. All plots were 50 m × 50 m, and flight paths followed a “Z”-shaped pattern based on plot boundaries [31], with detailed flight parameters shown in Table 1. UAV images were collected from all 72 plots, including 36 alpine meadow plots and 36 typical steppe plots. After image quality checking, the images were processed using Agisoft Metashape Professional software (version 2.2.2; Agisoft LLC, St. Petersburg, Russia) to generate orthomosaics. Vegetation and burrow metrics were subsequently derived from the orthomosaics.

In Table 1, forward overlap refers to the overlap between consecutive UAV images along the flight direction, while side overlap refers to the overlap between images acquired from adjacent flight lines.

After image quality checking, UAV images were processed using Agisoft Metashape Professional software (version 2.2.2; Agisoft LLC, St. Petersburg, Russia). The initial camera positions were obtained from the geotagged information recorded in the image metadata, including sensor location and flight altitude. Image alignment was then performed to identify homologous tie points among overlapping images. Based on these tie points, camera parameters and image positions were optimized, and a sparse point cloud was generated. A dense point cloud was subsequently generated from the sparse point cloud and used to produce a Digital Surface Model (DSM). Finally, orthomosaics, also referred to as Digital Orthophoto Maps (DOMs), were generated through orthorectification and texture mosaicking. Projection and coordinate system transformations were then conducted using ENVI 5.6 software.

Rodent burrow density was determined using custom-developed software based on the YOLOv5 object detection algorithm [32], implemented in a Python 3.7 environment (version 1.0; software registration number: 2021SR0546708). UAV images were preprocessed to meet model input requirements and then fed into the trained model for target detection. The final number of rodent burrows was obtained accordingly.

Vegetation cover and the visible-band difference vegetation index (VDVI) were extracted from UAV imagery. Vegetation cover was estimated using a neural network classification model in ENVI 5.6. VDVI was calculated from the red, green, and blue digital number values of the UAV RGB orthomosaics. To reduce illumination effects, flights were conducted under clear and windless conditions during similar daytime periods, and all images were processed using the same workflow. Previous studies have shown that VDVI is strongly positively correlated with aboveground biomass in grasslands and can effectively reflect vegetation growth status [33]. Therefore, VDVI was used as a key indicator for estimating aboveground biomass in this study. The calculation formula is as follows:

(4) $$VDVI=\frac{2\times DNgreen-DNred-DNblue}{2\times DNgreen+DNred+DNblue}$$

where DNgreen, $DN_{red}$, and $DN_{blue}$ represent the digital number values of the green, red, and blue bands in the UAV RGB imagery, respectively. VDVI was used as a continuous predictor variable in the aboveground-biomass estimation models rather than applying a fixed classification threshold. This approach retains the full range of vegetation greenness information captured by the index.

#### 2.2.3. Acquisition and Processing of Satellite Imagery

Landsat 8 imagery was selected for its free availability, open accessibility, and consistent revisit cycle, making it suitable for the space-based component of the integrated air–ground–space framework. All images were acquired through the Google Earth Engine (GEE) platform and temporally matched to the corresponding field surveys. Specifically, 16 scenes acquired during July–August 2021 were used for Ruoergai County, whereas 8 and 22 scenes acquired during July–August 2024 were used for Jiuzhi County and Dongwuzhumuqin Banner, respectively. These periods corresponded to the peak growing season and were aligned with the timing of UAV and ground-based data collection. Images from different years were processed separately for each study area and were not combined within the same regional composite. Cloud-contaminated and other low-quality pixels were removed using the quality assessment bands provided in the Landsat Collection 2 Level-2 products, and a pixel-wise median composite was generated for each study area to reduce the influence of cloud cover and improve data reliability. After cropping to the corresponding study-area boundaries and ensuring spatial consistency, the processed images were used to derive the Normalized Difference Vegetation Index (NDVI), Land Surface Temperature (LST), Wetness component (WET), and Normalized Difference Built-up and Bare Soil Index (NDBSI), which were subsequently integrated to construct the Remote Sensing Ecological Index (RSEI) (Table 2) [34].

### 2.3. Identification of Rodent Infestation Characteristics in Two Grassland Types Based on UAV Imagery

#### 2.3.1. Estimation of Aboveground Biomass for Two Grassland Types

In this study, VDVI was used as the independent variable and aboveground biomass as the dependent variable. Four univariate models, namely linear, quadratic polynomial, logarithmic, and exponential models, were constructed. The coefficient of determination ${(R}^{2}$) was used to evaluate the goodness of fit of each biomass inversion model, and the best-performing model was selected for estimating aboveground biomass in the two grassland types.

#### 2.3.2. Verification of Accuracy Between Ground Measurements and UAV Image Interpretations

This study employed linear regression analysis using field survey data on rodent burrow density, aboveground biomass, and vegetation cover, along with corresponding image interpretation data. Based on *p*-values, we assessed the statistical significance of each relationship and used adjusted${\;R}^{2}$ to evaluate model fit and the reliability of the image interpretation indicators.

### 2.4. Index Construction and Modeling

#### 2.4.1. Construction of the Rodent Damage Index (RDI)

First, the measured values of indicators such as aboveground biomass, community height, and vegetation cover were standardized using the maximum–minimum method to eliminate differences in units of measurement. Subsequently, principal component analysis (PCA) was employed to extract the principal components, and their weights were determined based on the variance contribution rate of each component. This process was used to construct RDI [35], calculated using the following formula:

(5) $$RDI=\sum_{k=1}^{m}\left(\frac{\lambda_{k}}{\sum_{i=1}^{m}\lambda_{i}}\right)\sum_{j=1}^{p}a_{jk}Z_{j}$$

where $\lambda_{k}$ is the eigenvalue corresponding to the kth principal component, $Z_{j}$ is the standardized value of the jth indicator, $a_{jk}$ is the corresponding principal component coefficient, and p is the number of original indicators.

#### 2.4.2. Calculation of the Remote Sensing Ecological Index (RSEI)

This study uses remote sensing imagery as its data foundation and selects typical remote sensing indicators with clear ecological significance to construct the RSEI, which is used to comprehensively evaluate the ecological and environmental quality of the study area. The construction of RSEI requires four indicators: NDVI, WET, NDBSI, and LST. The formula is as follows:

(6) RSEI=f{NDVI, WET, NDBSI, LST}

where f denotes principal component analysis.

First, four types of ecological indicators were calculated based on remote sensing imagery: In this study, NDVI serves as a direct remote sensing indicator of vegetation degradation following grassland damage caused by rodent disturbance; WET is used to reflect changes in surface moisture conditions and ecological water retention capacity under rodent disturbance; LST is used to characterize the extent of deterioration in the surface thermal environment following rodent disturbance; and NDBSI is used to remotely characterize the increased soil exposure and degradation caused by rodent disturbance. Its calculation formula is as follows:

(7) $$NDVI=\frac{\rho nir-\rho red}{\rho nir+\rho red}$$

where ρnir is the reflectance in the near-infrared band, and ρred is the surface reflectance in the visible red band.

(8) WET=0.1511ρ2+0.1973ρ3+0.3283ρ4+0.3407ρ5−0.7117ρ6−0.4559ρ7

where ρi (i = 1, 2, …, 7) represents the surface emissivity bands

(9) $$NDBSI=\frac{SI+IBI}{2}$$

(10) $$SI=\frac{\left(\rho 6+\rho 4\right)-\left(\rho 5+\rho 2\right)}{\left(\rho 6+\rho 4\right)+\left(\rho 5+\rho 2\right)}$$

(11) $$IBI=\frac{\frac{2\rho 6}{\rho 6+\rho 5}-\frac{\rho 5}{\rho 3+\rho 4}-\frac{\rho 3}{\rho 3+\rho 6}}{\frac{2\rho 6}{\rho 6+\rho 5}+\frac{\rho 5}{\rho 3+\rho 4}+\frac{\rho 3}{\rho 3+\rho 6}}$$

where ρi (i = 1, 2, …, 7) represents the surface emissivity bands

(12) $$LST={DN}_{ST\_B10}\times 0.00341802+149.0-273.15$$

where ${DN}_{ST\_B10}$ is the digital number of the Landsat 8 Collection 2 Level-2 ST-B10 band.

The aforementioned indicators reflect ecological conditions from four distinct perspectives. Among them, NDVI and WET serve as positive indicators of ecological conditions, while LST and NDBSI act as negative indicators. Furthermore, to eliminate the effects of differences in the units and numerical ranges of the various indicators, each individual indicator was normalized using the maximum–minimum method, thereby standardizing their value ranges to [0, 1]. Subsequently, PCA was applied to the standardized four indicators to perform dimensionality reduction and extract the first principal component. The first principal component typically explains most of the information contained in the original variables, and its eigenvector reflects the contribution of each ecological factor to the overall ecological environment quality. Finally, the first principal component was linearly normalized to construct RSEI, with a value range of [0, 1]. A higher RSEI value indicates better regional ecological environment quality [36]. By analyzing the spatial distribution characteristics of the RSEI, the spatial patterns of ecological environment quality differences and their variation characteristics caused by rodent disturbance in the study area’s natural grasslands can be intuitively revealed.

#### 2.4.3. Development of the Assessment Model

To support regional estimation of rodent damage severity, the relationship between plot-scale RDI and RSEI was modeled separately for alpine meadows and typical steppe using ordinary least-squares linear regression. This approach was selected because of its simplicity, interpretability, and suitability for spatial extrapolation. Given the limited sample sizes, a fixed training–test split was not adopted because it would have further reduced the number of observations available for model development and produced results that were highly dependent on a single data partition. Instead, leave-one-out cross-validation (LOOCV) was used to evaluate predictive performance. In each iteration, one observation was withheld as the validation sample, and the model was fitted using the remaining n−1 observations. The fitted model was then used to predict the RDI of the withheld observation. This procedure was repeated until each observation had served once as the validation sample. The LOOCV ${\;R}^{2}$ and RMSE were calculated from the pooled predictions for all withheld observations. In addition, fitting${\;R}^{2}$ and RMSE were calculated from models fitted to all valid observations and were reported only as measures of model fit, whereas the LOOCV metrics were used to assess predictive performance. After validation, the final regression models were refitted using all valid observations and applied to regional RSEI data to generate the spatial rodent damage risk maps.

The linear regression model was expressed as follows:

(13) RDI=kRSEI+b

where k is the regression coefficient and b is the intercept.

**Figure 2:**
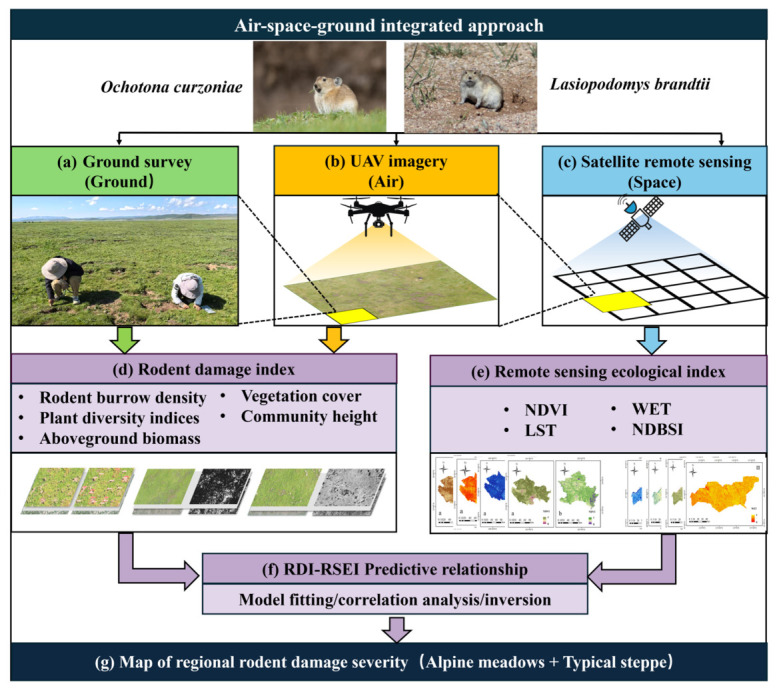
Air–space–ground integrated framework for rodent damage monitoring and regional mapping.

The resulting models were then applied to the RSEI raster layers to generate continuous spatial maps of predicted RDI across the entire study area. Higher RDI values indicate more severe overall rodent damage, encompassing reduced vegetation biomass, cover, height, diversity, and increased burrow density. This approach enables regional-scale assessment of rodent damage severity based on readily available satellite data. The overall framework is shown in Figure 2.

## 3. Results

### 3.1. Identification of Rodent Damage Severity Characteristics in Two Grassland Types Based on UAV Imagery

#### 3.1.1. Estimated Aboveground Biomass for Two Grassland Types

**Figure 3:**
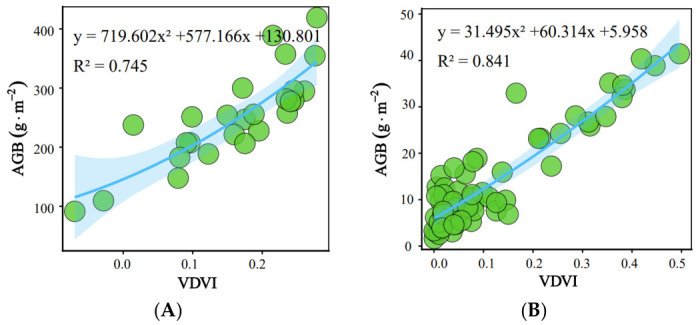
Aboveground biomass inversion model. Note: (**A**) alpine meadows, (**B**) typical steppe.

After comparison, a quadratic polynomial regression model was selected as the optimal inversion model for the two grassland types (Table 3), and the model was expressed as follows(Figure 3):

(14) $$y_{a}=719.602{x_{a}}^{2}+577.166x_{a}+130.801$$

(15) $$y_{b}=31.495{x_{b}}^{2}+60.314x_{b}+5.958$$

where y represents ground-measured grassland aboveground biomass and x represents VDVI in the visible light spectrum.

**Figure 4:**
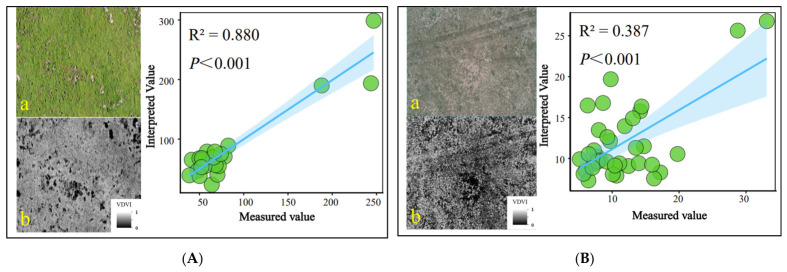
Correlation between measured and predicted aboveground biomass. Note: (**A**) alpine meadows, (**B**) typical steppe. For each image: (**a**) original UAV image, (**b**) interpreted image.

Following model development, validation was conducted by comparing the observed aboveground biomass with the model-estimated values, computing the Mean Relative Error (MRE) and coefficient of determination ($R^{2}$). For alpine meadows, the model demonstrated reasonable accuracy with an MRE = 0.196 and $R^{2}$ = 0.880 (Figure 4A). For typical steppe, the model showed an MRE = 0.377 and $R^{2}$ = 0.387 (Figure 4B). The model performed considerably better in alpine meadows than in typical steppe.

#### 3.1.2. Rodent Burrow Density Detection

**Figure 5:**
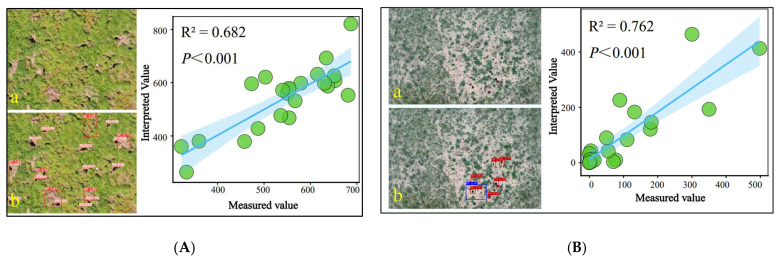
Correlation between measured and predicted rodent burrow density. Note: (**A**) alpine meadows, (**B**) typical steppe. For each image: (**a**) original UAV image, (**b**) interpreted image.

For the validation of UAV-based burrow entrance detection, approximately 30% of the 36 plots in each grassland type were selected using stratified random sampling to ensure coverage of the observed range of burrow entrance densities. UAV-interpreted burrow entrance counts were then compared with the corresponding field-observed counts. Based on the validation plots, UAV-based burrow entrance detection showed different accuracy between the two grassland types. In alpine meadows, the detection accuracy was relatively high, MRE = 0.106 and $R^{2}$ = 0.682 (Figure 5A). In typical steppe, MRE = 0.362 and $R^{2}$ = 0.762 (Figure 5B), indicating that although the detected and observed burrow numbers were correlated, the relative error was higher than that in alpine meadows.

#### 3.1.3. Vegetation Cover Recognition

**Figure 6:**
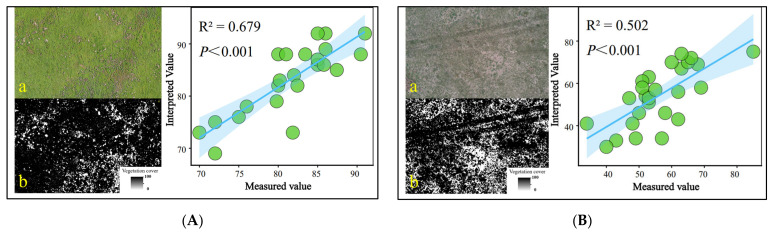
Correlation between measured and predicted vegetation cover. Note: (**A**) alpine meadows, (**B**) typical steppe. For each image: (**a**) original UAV image, (**b**) interpreted image.

The validation results are as follows: for alpine meadows, MRE = 0.039 and $R^{2}$ = 0.679 (Figure 6A); for typical steppe, MRE = 0.148 and $R^{2}$ = 0.502 (Figure 6B). These results indicate that UAV-based vegetation cover extraction performed well in alpine meadows but showed only moderate accuracy in typical steppe. Therefore, vegetation cover estimates for typical steppe should be interpreted with caution.

### 3.2. Construction of the RDI

#### 3.2.1. Construction of RDI for Alpine Meadows

Principal component analysis was applied to the damage assessment indicators for alpine meadows. As shown in Table 4, the cumulative variance contribution of the first three principal components reached 80.80%, indicating that PC1–PC3 captured the dominant variation among the original indicators, including rodent burrow density, aboveground biomass, vegetation cover, community height, the Shannon–Wiener diversity index, and the Margalef index. Therefore, these three principal components were retained for RDI construction. Although PC4 explained an additional 9.27% of the variance, its eigenvalue was lower than 1 and its contribution was relatively small compared with the first three components. Thus, PC4 was not included in the RDI formula to avoid introducing weak and potentially unstable components into the weighting scheme.

We calculated the scores of the first three principal components using the corresponding component-score coefficients as follows:

(16) $$PC1=0.360\times AGB+0.452\times VC+0.452\times CH-0.353D-0.408H^{\prime }-0.414RBD$$

(17) $$PC2=0.497\times AGB+0.420\times VC+0.245\times CH+0.433D+0.486H^{\prime }+0.306RBD$$

(18) $$PC3=-0.441\times AGB-0.061\times VC+0.523\times CH+0.656D-0.182H^{\prime }-0.255RBD$$

Here, PC1, PC2, and PC3 represent the first three principal components, respectively. AGB, VC, CH, $H^{\prime }$, D, and RBD represent the standardized values of aboveground biomass, vegetation cover, community height, Shannon–Wiener diversity index, the Margalef index, and rodent burrow density, respectively. The contribution rates of each indicator to the damage assessment index are calculated using the weights derived from the eigenvalues of the three principal components (Table 5). RDI for alpine meadow plateau pikas is then calculated as follows:

(19) RDI=0.5511×PC1+0.3093×PC2+0.1403×PC3

#### 3.2.2. Development of the Typical Steppe RDI

Principal component analysis was applied to the established indicators for assessing damage in typical steppe. As shown in Table 6, the cumulative variance explained by the first three principal components reaches 88.56%. Therefore, the damage characteristics of Brandt’s vole can essentially be reflected by these first three principal components, and the original variables—rodent burrow density, aboveground biomass, vegetation cover, community height, Shannon–Wiener diversity index and the Margalef index—can be replaced by these three principal components.

We calculated the scores of the first three principal components using the corresponding component-score coefficients as follows:

(20) $$PC1=0.214\times CH+0.170\times VC+0.221\times AGB-0.012RBD+0.251H^{\prime }++0.259D$$

(21) $$PC2=-0.265\times CH-0288\times VC+0.269\times AGB+0.293RBD+0.259H^{\prime }+0.203D$$

(22) $$PC3=0.013\times CH+0.317\times VC+0.279\times AGB+0.943RBD-0.134H^{\prime }+0.056D$$

Here, PC1, PC2, and PC3 represent the first three principal components, respectively. AGB, VC, CH, $H^{\prime }$, D, and RBD represent the standardized values of aboveground biomass, vegetation cover, community height, Shannon–Wiener diversity index, the Margalef index, and rodent burrow density, respectively. The contribution rates of each indicator to the damage assessment index are calculated using the initial eigenvalue weights of the three principal components (Table 7). RDI for a typical steppe is then calculated as follows:

(23) RDI=0.5249×PC1+0.3441×PC2+0.1309×PC3

### 3.3. Development of RSEI

#### 3.3.1. Development of RSEI for Alpine Meadows

**Figure 7:**
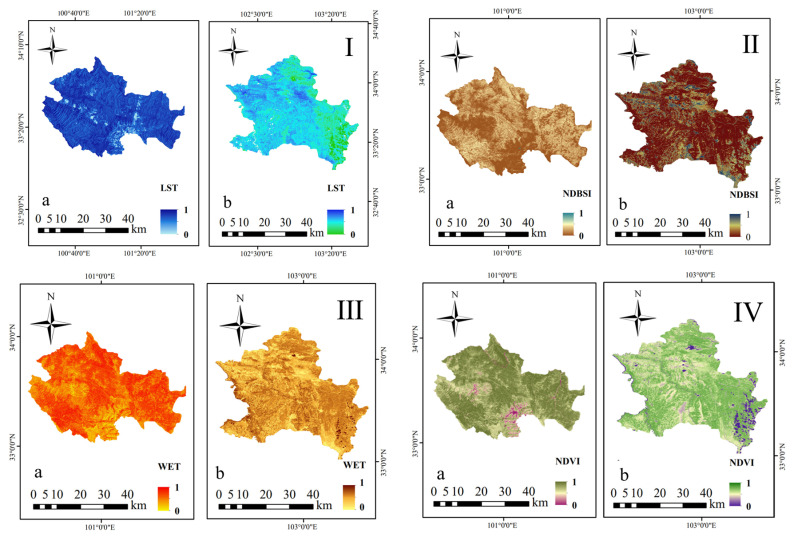
Spatial distribution of the component indicators of RSEI. Note: (**I**) LST, (**II**) NDBSI, (**III**) WET, (**IV**) NDVI. For each indicator, panels (**a**,**b**) represent two sub-regions within the alpine meadow study area.

Using Landsat imagery, four regional-scale ecological indicators (NDVI, WET, NDBSI, and LST) were extracted for the alpine meadow study area (Figure 7). The spatial distribution of these indices reveals distinct differences between sub-study areas (a) and (b). Regarding abiotic factors, LST in area (a) displays a relatively continuous distribution, whereas area (b) exhibits pronounced thermal patchiness. Similarly, while both areas show moderate-to-high NDBSI values, area (b) presents a broader range of high values with greater spatial fluctuation.

For biotic and moisture conditions, area (a) maintains a high and uniform NDVI. In contrast, area (b) contains numerous scattered low-value NDVI patches. Furthermore, WET shows localized reductions in surface moisture within area (b).

Overall, area (a) is characterized by a uniform spatial distribution across vegetation, thermal, and moisture conditions. Conversely, area (b) demonstrates notably higher spatial heterogeneity, characterized by elevated bare soil exposure and fragmented vegetation cover.

**Figure 8:**
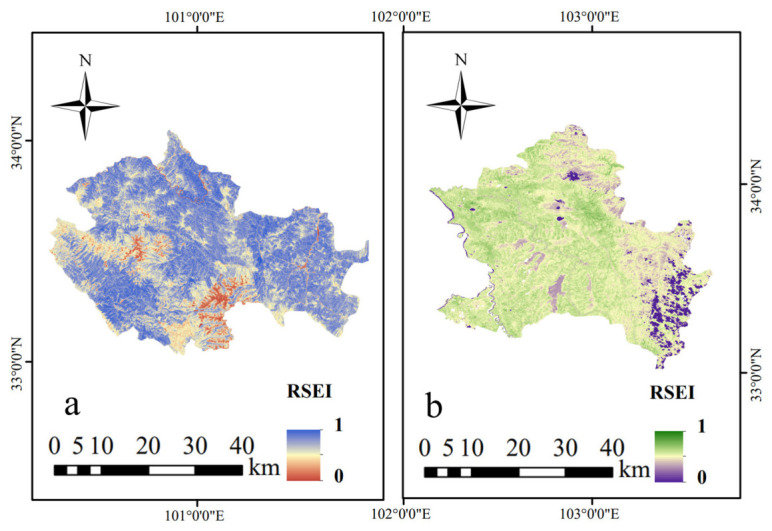
Spatial distribution of RSEI. Note: panels (**a**,**b**) represent two sub-regions within the alpine meadow study area.

The spatial distribution of RSEI (Figure 8) revealed clear heterogeneity in ecological quality across the alpine meadow study area. In area (a), high RSEI values were distributed relatively continuously, whereas low values occurred mainly in scattered patches. The mean RSEI = 0.749 ± 0.093, with values ranging from 0 to 1. In area (b), high-value areas were interspersed with clustered low-value patches, and the mean RSEI = 0.701 ± 0.099, also ranging from 0 to 1. Overall, area (a) had a higher mean RSEI, indicating relatively better ecological conditions, whereas area (b) exhibited slightly greater spatial variability, as reflected by its higher standard deviation and more clustered distribution of low-RSEI patches. This spatial pattern broadly corresponded to the observed heterogeneity in plateau pika disturbance.

#### 3.3.2. Development of RSEI for Typical Steppe

**Figure 9:**
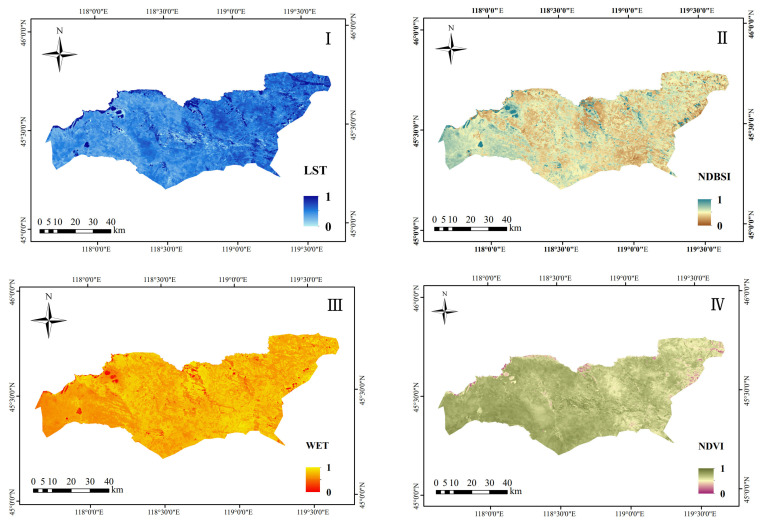
Spatial distribution of the component indicators of RSEI in the typical steppe. Note: (**I**) LST, (**II**) NDBSI, (**III**) WET, (**IV**) NDVI.

Using Landsat imagery, four regional-scale ecological indicators (NDVI, WET, NDBSI, and LST) were extracted for the typical steppe study area (Figure 9). The spatial patterns of these indices showed clear heterogeneity across the study area. LST and NDBSI exhibited relatively higher values in some localized patches, indicating increased surface exposure and thermal stress. In contrast, NDVI remained generally high, although scattered low-value patches were present, while WET was relatively uniform with localized reductions in surface moisture. Overall, areas with higher LST and NDBSI and lower NDVI and WET were more likely to reflect localized ecological deterioration under Brandt’s vole disturbance.

**Figure 10:**
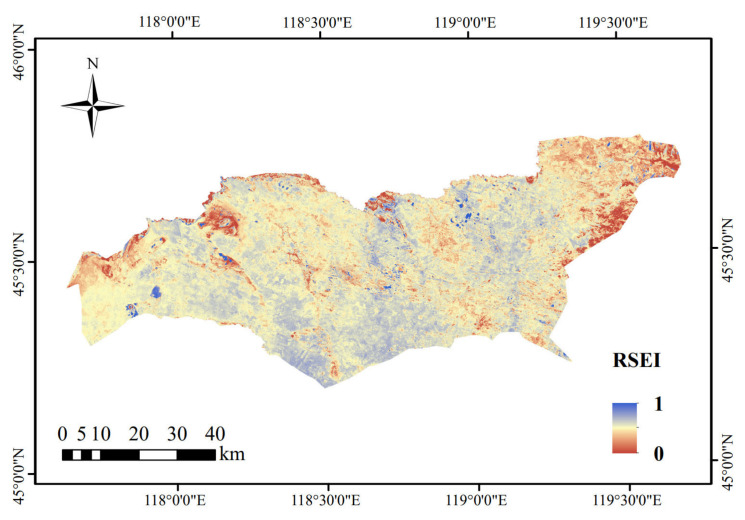
Spatial distribution of RSEI in the typical steppe.

The spatial distribution of RSEI values (Figure 10) reveals significant spatial variations in the ecological and environmental quality of the study area. Overall, the central regions of the study area exhibit relatively high RSEI values and better ecological and environmental quality, indicating good vegetation cover, higher surface humidity, and a relatively stable thermal environment in these areas. In contrast, some peripheral regions have relatively low RSEI values and poorer ecological and environmental quality, often characterized by low vegetation cover and higher surface temperatures. To some extent, this may reflect the spatial response to vegetation degradation, the expansion of bare ground, and the deterioration of the surface environment under the influence of Brandt’s vole.

### 3.4. Modeling RDI and RSEI

**Figure 11:**
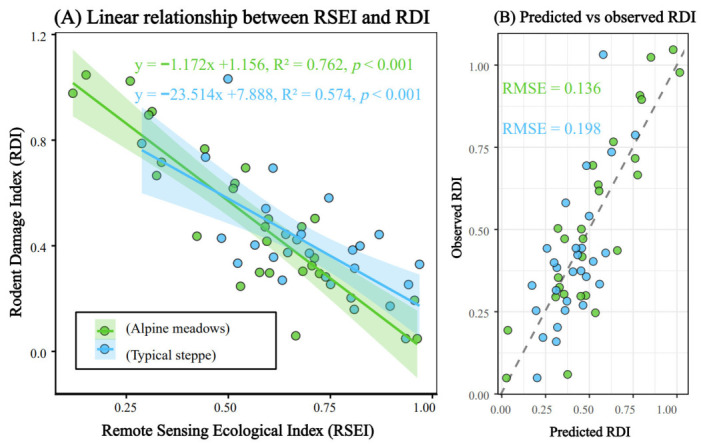
Grassland-type-specific linear regression models linking RDI and RSEI. (**A**) Relationships between plot-scale Rodent Damage Index (RDI) and satellite-derived Remote Sensing Ecological Index (RSEI) in alpine meadows and typical steppe. (**B**) Observed versus predicted RDI values. The dashed line indicates the 1:1 line, and RMSE values represent model prediction errors.

RDI was constructed using vegetation indices derived from ground-based vegetation survey data and UAV imagery, and compared with the RSEI derived from satellite remote sensing imagery. The results indicate that there is a certain correlation between RDI and RSEI (Figure 11). In particular, the fit between RDI and RSEI was better in the alpine meadows, $R^{2}$ = 0.762 and RMSE = 0.136, while the LOOCV results showed a cross-validated $R^{2}$ = 0.729 and RMSE = 0.145, 95% CI: 0.580–0.886. indicating a strong response relationship between RDI and high RSEI values. In a typical steppe, the RDI–RSEI relationship showed lower explanatory power than that in alpine meadows. The fitted linear regression model had an $R^{2}$ = 0.574 and RMSE of 0.198, while the LOOCV results showed a cross-validated $R^{2}$ = 0.478 and RMSE = 0.214, 95% CI: 0.279–0.787. This indicates that a considerable proportion of RDI variation in typical steppe was not explained by RSEI alone.

### 3.5. Model Inversion

**Figure 12:**
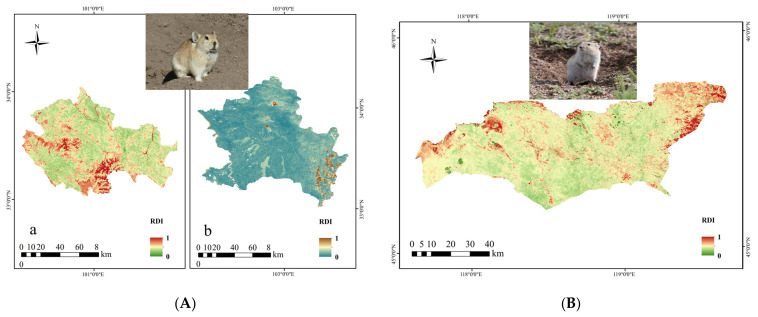
Regional-scale inversion results of the Rodent Damage Index. (**A**) Alpine meadow area, with sub-regions (**a**) and (**b**) illustrating contrasting spatial patterns of plateau pika damage; (**B**) Typical steppe area, showing the spatial distribution of Brandt’s vole damage severity.

Based on the established RDI–RSEI inversion model, RSEI values for each pixel in the study area were derived using Landsat 8 remote sensing imagery. These values were then substituted into the established RDI-RSEI inversion model to calculate the spatial distribution of the Regional RDI at the regional scale. The inversion results indicate that rodent damage severity in the study area exhibits distinct spatial heterogeneity, with significant differences in damage levels across different regions. Results from both alpine meadows and typical steppe suggest that rodent damage severity is significantly correlated with the regional ecological conditions (Figure 12).

## 4. Discussion

### 4.1. Monitoring Rodent Damage Severity in Natural Grasslands Using Multi-Source Data

The main contribution of this study is not simply demonstrating that UAV imagery can detect surface changes, as this capability has been widely reported in previous grassland monitoring studies [37]. Instead, this study used UAV-derived information as an intermediate scale to connect field-based ecological measurements with satellite-based regional assessment. Ground surveys provided reliable plot-scale measurements of vegetation and rodent disturbance. UAV imagery was suitable for plot- and local-scale monitoring because it captured fine-scale spatial features such as burrow openings, vegetation cover, and aboveground biomass variation within the 50 m × 50 m plots. In contrast, satellite remote sensing was suitable for regional-scale monitoring because it provided continuous spatial coverage over larger areas [38]. These UAV-derived indicators helped translate field observations into spatially explicit plot-level damage information and supported the subsequent linkage with satellite-derived ecological conditions.

By further linking the plot-scale Rodent Damage Index (RDI) with the satellite-derived Remote Sensing Ecological Index (RSEI), this study established a cross-scale framework for regional rodent damage assessment. Compared with studies that focus only on UAV-based feature extraction or satellite-based vegetation degradation monitoring, the present framework integrates ground, UAV, and satellite data into a unified assessment pathway [18,39]. This linkage is important because rodent damage is expressed through multiple ecological responses, including reduced vegetation biomass and cover, altered surface moisture, increased bare soil exposure, and changes in surface thermal conditions. Therefore, the RDI–RSEI relationship provides a practical way to extend field-based damage assessment to larger spatial scales.

From a management perspective, this framework can help identify potential high-risk areas of rodent disturbance and support targeted rodent damage management planning. However, the framework should be interpreted as a regional risk-assessment tool rather than a fully validated operational product, especially in areas where independent field validation data are limited.

### 4.2. Differences in Remote Sensing Responses to Rodent Damage Severity Across Different Grassland Types

The performance of rodent damage monitoring differed markedly between grassland types, indicating that remote sensing responses to rodent disturbance are strongly influenced by ecological context. The RDI–RSEI relationship was stronger in alpine meadows than in typical steppe, suggesting that the same remote sensing index may not capture rodent damage with equal effectiveness across different grassland ecosystems [40]. This result highlights the necessity of grassland-type-specific modeling rather than applying a single generalized model to all natural grasslands.

One plausible explanation for the better model performance in alpine meadows is the difference in vegetation background and disturbance expression between the two grassland types. Alpine meadows generally have relatively dense and continuous vegetation cover, so plateau pika burrowing may generate more conspicuous exposed soil patches, vegetation fragmentation, and biomass reduction, which can be more readily reflected by composite remote sensing indices such as RSEI [41,42]. In contrast, a typical steppe is characterized by sparser vegetation, stronger bare-ground background, and a generally yellowish-brown surface tone. Under such conditions, burrow disturbance signals may be more easily mixed with naturally exposed soil, drought-induced vegetation variation, litter, shadows, and other environmental noise, reducing the distinctness of remote sensing responses to rodent disturbance [36]. However, this explanation should be regarded as a plausible hypothesis rather than direct evidence, because this study did not quantitatively test the relationship between model residuals and vegetation density metrics such as vegetation cover, aboveground biomass, or community height.

The validation of burrow entrance extraction further illustrates the influence of grassland background on monitoring accuracy. In alpine meadows, the lower MRE indicated better quantitative agreement between interpreted and observed burrow entrance numbers. In a typical steppe, the higher $R^{2}$ but markedly larger MRE suggested that the interpretation results were better at capturing relative differences among plots than at accurately estimating absolute burrow entrance numbers. This pattern is not contradictory, because $R^{2}$ mainly reflects the consistency of variation trends, whereas MRE emphasizes the magnitude of relative deviation in absolute counts. The higher background heterogeneity in a typical steppe may have increased both omission and commission errors, thereby weakening quantitative precision while still preserving the relative ranking of burrow abundance among plots [43,44].

The relatively low contribution of burrow density to the final RDI in the typical steppe also reflects the complexity of Brandt’s vole disturbance. Although burrow density is an important field indicator of rodent activity, Brandt’s vole damage is not expressed only through burrowing. Population outbreaks and foraging activities can directly reduce vegetation cover, aboveground biomass, and community height, while the naturally sparse vegetation background of a typical steppe may weaken the remote sensing signal of burrow openings [45]. Therefore, the RDI for typical steppe should be interpreted as a composite index of overall rodent damage severity rather than a burrow-density-dominated index. Moreover, the lower explanatory power of the RDI–RSEI model in a typical steppe indicates that RSEI alone cannot fully capture the complexity of Brandt’s vole disturbance. Factors such as livestock grazing intensity, precipitation variability, soil texture, and local topography may also influence vegetation cover, surface moisture, and bare soil exposure, thereby contributing to the unexplained variation in RDI [46,47]. Because these factors were not included as covariates in the model, the RDI–RSEI relationship cannot isolate the independent effect of rodent disturbance on RSEI. Accordingly, the regional mapping results, especially for typical steppe, should be interpreted as potential rodent damage risk patterns under combined ecological influences, rather than as causal maps of rodent-induced degradation or fully validated damage products. Despite these uncertainties, the type-specific framework proposed in this study can still provide useful support for the rapid identification of potential high-risk areas and regional rodent damage management planning.

### 4.3. Implications for Wildlife or Habitat Management

From a wildlife and habitat management perspective, the main contribution of this study is providing a remote sensing-based framework for identifying areas with a high risk of rodent-induced grassland disturbance without relying solely on extensive field surveys. In this context, high-risk areas are not only characterized by high rodent activity, but also by associated ecological changes such as loss of vegetation cover, increased bare soil exposure, reduced aboveground biomass, and changes in surface heterogeneity. These changes may alter habitat structure and habitat complexity, which are important for both small mammals and other grassland-associated wildlife. For prey species such as plateau pika and Brandt’s vole, changes in vegetation cover and bare-ground exposure may also influence visibility, refuge availability, and predation risk, thereby affecting predator–prey interactions in grassland ecosystems.

The proposed ground–UAV–satellite framework enables managers to map potential rodent damage severity across regional scales and prioritize field monitoring or management actions in areas where ecological degradation is most evident. UAV imagery provides fine-scale information on burrow openings, vegetation cover, and surface disturbance within plots, whereas satellite-derived RSEI allows broader regional assessment of ecological conditions. By distinguishing between alpine meadows and typical steppe, the results also indicate that management strategies should be grassland-type-specific rather than applying a uniform approach across different grassland ecosystems.

Beyond rodent damage assessment, this cross-scale monitoring framework may also be useful for broader wildlife conservation and habitat management applications. For example, similar approaches could be applied to monitor habitat degradation, vegetation loss, grazing impacts, bare-ground expansion, and the spatial recovery of degraded grasslands after management interventions. In ecologically fragile regions such as the Qinghai–Tibet Plateau and Inner Mongolia, this framework can support early warning of grassland degradation, guide targeted field surveys, and provide spatial information for adaptive grassland and wildlife habitat management.

### 4.4. Limitations of This Study

This study has several limitations. First, the regional rodent damage maps lacked independent field validation. Although the RDI–RSEI relationships were evaluated using plot-scale samples and leave-one-out cross-validation, no independent field survey sites outside the model-development dataset were available to verify the accuracy of the final regional maps. Therefore, the mapped results should be interpreted as potential spatial patterns of rodent damage risk rather than fully validated operational products. This limitation is particularly important for the typical steppe, where the model explained only 55.9% of the variation in RDI, leaving 44.1% unexplained. Accordingly, the typical steppe map should be regarded as a preliminary spatial screening product for identifying areas that may require priority field inspection, rather than as a definitive map of rodent damage severity. Local management decisions should therefore be supported by additional field observations.

Second, RSEI is a composite indicator of regional ecological condition rather than a rodent-specific disturbance index. Although it integrates vegetation greenness, surface moisture, surface dryness, and land surface temperature, it is also influenced by climate variability, livestock grazing, soil properties, vegetation phenology, and topographic conditions. These potential confounding factors were not explicitly controlled for in the RDI–RSEI models because spatially matched data on grazing intensity, precipitation variability, soil characteristics, and topography were not consistently available across all plots and mapping areas. Therefore, the observed relationship between RDI and RSEI should be interpreted as an association between plot-scale rodent damage severity and regional ecological condition, rather than as direct causal evidence that changes in RSEI were caused solely by rodent disturbance. This issue is especially relevant in the typical steppe, where sparse vegetation, strong bare-soil background effects, grazing disturbance, and precipitation variability may jointly affect RSEI values. Future studies should incorporate additional covariates, including grazing intensity, precipitation, soil texture and fertility, elevation, slope, and other topographic variables, into multivariable or spatial models to better distinguish rodent-induced disturbance from other environmental drivers.

Third, although the ground observations, UAV-derived indicators, and Landsat-based RSEI were spatially matched at the plot level, some differences in spatial support and resolution remained among the three data sources. Ground measurements were obtained from relatively small quadrats or sampling points and then aggregated to represent each 50 m × 50 m plot, whereas UAV imagery captured fine-scale spatial variation at centimeter resolution, and Landsat pixels represented ecological conditions at a 30 m resolution. In addition, plot boundaries did not necessarily coincide exactly with the Landsat pixel grid, which may have introduced mixed-pixel and aggregation effects. These scale differences may have contributed to uncertainty during the conversion from plot-level observations to regional assessment. Future studies should further improve spatial co-registration and temporal consistency and evaluate multiscale aggregation methods.

Fourth, the UAV RGB imagery used in this study was not radiometrically calibrated with a reflectance panel. Consequently, VDVI values were derived from RGB digital numbers rather than absolute surface reflectance. Although UAV flights were conducted under broadly similar weather and illumination conditions and all images were processed using a consistent workflow, residual differences in illumination among flights may have affected the comparability of UAV-derived vegetation indices. Future studies should use reflectance panels or radiometrically calibrated sensors to improve the reliability and comparability of UAV-based vegetation indicators.

Finally, this study focused on only two representative natural grassland types, namely alpine meadows and typical steppe. The applicability of the proposed framework to other grassland ecosystems, geographic regions, and rodent species remains uncertain. Future research should expand the sampling scope, include a wider range of grassland types and disturbance gradients, and conduct independent field validation across multiple regions to improve the robustness, transferability, and operational applicability of the proposed monitoring framework.

## 5. Conclusions

This study constructed a plot-scale RDI using ground survey and UAV-derived indicators and linked it with the satellite-derived RSEI to explore the regional-scale assessment of rodent damage severity in natural grasslands. The results showed that RDI and RSEI were negatively correlated in both alpine meadows and typical steppe, but model performance differed markedly between the two grassland types. The linear regression model performed better in alpine meadows, with a fitting $R^{2}$ = 0.762 and a LOOCV $R^{2}$ = 0.729, whereas the model showed weaker performance in typical steppe, with a fitting $R^{2}$ = 0.574 and a LOOCV $R^{2}$ of 0.478. These results indicate that remote sensing responses to rodent disturbance vary with ecological context and that grassland-type-specific modeling is necessary for regional rodent damage assessment.

Overall, the integration of ground survey data, UAV-derived fine-resolution information, and satellite-based regional observation provides a preliminary cross-scale framework for identifying potential rodent damage risk areas in natural grasslands. However, before this framework can be deployed as an operational monitoring tool, several issues need to be addressed. First, independent field validation plots should be used to verify the accuracy of the regional damage maps, especially across different damage levels and environmental backgrounds. Second, multi-year and multi-season observations are needed to test the temporal stability of the RDI–RSEI relationship under varying climate and grazing conditions. Third, the framework should be further evaluated in other grassland types, regions, and rodent species to improve its transferability and general applicability. Therefore, the current mapping results should be interpreted as potential rodent damage risk patterns rather than fully validated operational products.

## Figures and Tables

**Table 1:** UAV Flight Parameters.

Parameters	Value	Parameters	Value
Flight altitude	20 m	Field of View	84°
Flight speed	1.5 m/s	Ground Resolution	1.0 cm/pixel
Heading overlap	75%	Image Format	JPEG
Sideways overlap	75%	Shooting Angle/Direction	view from above

Note: Heading overlap, also referred to as forward overlap, indicates the percentage of overlap between consecutive images along the flight direction. Sideways overlap, also referred to as side overlap, indicates the percentage of overlap between images from adjacent flight lines.

**Table 2:** Remote sensing indicators derived from Landsat 8 imagery.

Sensor Types	Index	Interpretation of the Index	Using Bands	Resolution
Landsat8-OLI	NDVI	Primarily reflects vegetation growth status, coverage, and biological activity.	B4, B5	30 m
Landsat8-OLI	NDBSI	Primarily indicates bare soil and degraded land surface conditions.	B2, B4, B5, B6, B7	30 m
Landsat8-OLI	WET	Primarily reflects surface moisture characteristics and is closely associated with soil and vegetation water content.	B2–B7	30 m
Landsat8-TIRS	LST	Primarily reflects the thermal condition of the land surface.	B10 (±B11)	30 m

Note: LST was derived from the Landsat 8 TIRS thermal infrared band using the thermal conversion formula described in Equation (12). The final LST values were expressed in °C.

**Table 3:** Aboveground Biomass Inversion Models for Alpine Meadows and Typical Steppe.

Grassland Type	Estimation Model	Estimation Equation	R^2^	F	Sig
Alpine meadows	Linear Model	$y_{a}=755.10x_{a}+126.495$	0.738	67.208	0.000
	Quadratic Model	$y_{a}=719.602x_{a}^{2}+577.166x_{a}+130.801$	0.745	33.615	0.000
	Exponential Model	$y_{a}=138.164e^{3.385x_{a}}$	0.743	543.074	0.000
	Logarithmic Model	$y_{b}=126.265\ln\left(x_{a}\right)+487.014$	0.583	329.466	0.000
Typical steppe	Linear Model	$y_{b}=73.111x_{b}+5.447$	0.839	343.259	0.000
	Quadratic Model	$y_{b}=31.495x_{b}^{2}+60.314x_{b}+5.958$	0.841	171.648	0.000
	Exponential Model	$y_{b}=6.183e^{4.6261x_{b}}$	0.641	117.821	0.000
	Logarithmic Model	$y_{b}=4.744\ln\left(x_{b}\right)+27.974$	0.508	68.195	0.000

**Table 4:** Principal component eigenvalues and variance contribution rates for RDI construction in alpine meadows.

Component	Initial Eigenvalue	Contribution Rate (%)	Cumulative Variance (%)
1	2.669	44.48	44.48
2	1.499	24.99	69.47
3	0.680	11.33	80.80
4	0.556	9.27	90.07
5	0.323	5.38	95.46
6	0.272	4.54	100.00

**Table 5:** PCA loading coefficients and score coefficients for RDI construction in alpine meadows.

Indicator	First PC	Second PC	Third PC	First PC Coef	Second PC Coef	Third PC Coef
AGB	0.589	0.609	−0.364	0.360	0.497	−0.441
VC	0.738	0.514	−0.050	0.452	0.420	−0.061
CH	0.738	0.300	0.431	0.452	0.245	0.523
D	−0.577	0.530	0.541	−0.353	0.433	0.656
$H^{\prime }$	−0.666	0.595	−0.150	−0.408	0.486	−0.182
RBD	−0.676	0.375	−0.210	−0.414	0.306	−0.255

**Table 6:** Principal component eigenvalues and variance contribution rates for RDI construction in typical steppe.

Component	Initial Eigenvalue	Contribution Rate (%)	Cumulative Variance (%)
1	3.254	46.48	46.48
2	2.134	30.48	76.96
3	0.812	11.60	88.56
4	0.425	6.08	94.64
5	0.221	3.15	97.79
6	0.139	1.98	100.00

**Table 7:** PCA loading coefficients and score coefficients for RDI construction in a typical steppe.

Indicator	First PC	Second PC	Third PC	First PC Coef	Second PC Coef	Third PC Coef
CH	0.695	−0.565	0.011	0.214	−0.265	0.013
VC	0.553	−0.614	0.258	0.170	−0.288	0.317
AGB	0.719	−0.576	0.227	0.221	0.269	0.279
RBD	−0.039	0.626	0.765	−0.012	0.293	0.943
$H^{\prime }$	0.818	0.554	−0.109	0.251	0.259	−0.134
D	0.843	0.434	0.045	0.259	0.203	0.056

## Data Availability

The datasets used and/or analyzed during the current study are available from the corresponding author on reasonable request.

## Abbreviations

The following abbreviations are used in this manuscript:

UAV	Unmanned Aerial Vehicle
RDI	Rodent Damage Index
RSEI	Remote Sensing Ecological Index
LST	Land Surface Temperature
NDBSI	Normalized Difference Built-up and Bare Soil Index
WET	Wetness component
NDVI	Normalized Difference Vegetation Index
DSM	Digital Surface Model
DOM	Digital Orthophoto Map
VDVI	Visible-band Difference Vegetation Index
PCA	Principal Component Analysis
